# Control of Brain State Transitions with a Photoswitchable Muscarinic Agonist

**DOI:** 10.1002/advs.202005027

**Published:** 2021-05-21

**Authors:** Almudena Barbero‐Castillo, Fabio Riefolo, Carlo Matera, Sara Caldas‐Martínez, Pedro Mateos‐Aparicio, Julia F. Weinert, Aida Garrido‐Charles, Enrique Claro, Maria V. Sanchez‐Vives, Pau Gorostiza

**Affiliations:** ^1^ Institut d'Investigacions Biomèdiques August Pi i Sunyer (IDIBAPS) Barcelona 08036 Spain; ^2^ Institute for Bioengineering of Catalonia (IBEC) The Barcelona Institute for Science and Technology Barcelona 08028 Spain; ^3^ Network Biomedical Research Center in Bioengineering Biomaterials, and Nanomedicine (CIBER‐BBN) Madrid 28029 Spain; ^4^ Department of Pharmaceutical Sciences University of Milan Milan 20133 Italy; ^5^ Institut de Neurociències and Departament de Bioquímica i Biologia Molecular Unitat de Bioquímica de Medicina Universitat Autònoma de Barcelona (UAB) Barcelona 08193 Spain; ^6^ Catalan Institution for Research and Advanced Studies (ICREA) Barcelona 08010 Spain

**Keywords:** brain states, light‐mediated control, muscarinic acetylcholine receptors, neuromodulation, photopharmacology

## Abstract

The ability to control neural activity is essential for research not only in basic neuroscience, as spatiotemporal control of activity is a fundamental experimental tool, but also in clinical neurology for therapeutic brain interventions. Transcranial‐magnetic, ultrasound, and alternating/direct current (AC/DC) stimulation are some available means of spatiotemporal controlled neuromodulation. There is also light‐mediated control, such as optogenetics, which has revolutionized neuroscience research, yet its clinical translation is hampered by the need for gene manipulation. As a drug‐based light‐mediated control, the effect of a photoswitchable muscarinic agonist (Phthalimide‐Azo‐Iper (PAI)) on a brain network is evaluated in this study. First, the conditions to manipulate M2 muscarinic receptors with light in the experimental setup are determined. Next, physiological synchronous emergent cortical activity consisting of slow oscillations—as in slow wave sleep—is transformed into a higher frequency pattern in the cerebral cortex, both in vitro and in vivo, as a consequence of PAI activation with light. These results open the way to study cholinergic neuromodulation and to control spatiotemporal patterns of activity in different brain states, their transitions, and their links to cognition and behavior. The approach can be applied to different organisms and does not require genetic manipulation, which would make it translational to humans.

## Graphical abstract

Brain pathologies often require drug treatments, however drugs act all over the central nervous system. Wouldn't it be good to determine where/when a drug should be active? Drugs can be made sensitive to light, to be activated at specific times and locations. This study demonstrates that a light‐activated cholinergic drug can effectively modulate activity in the cerebral cortex network.

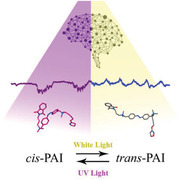

## 1 Introduction

All perceptions, memories, and behaviors are based on the communication between the billions of neurons that constitute the brain.^[^ ^1^, ^2^, ^3^, ^4^ ^]^ Individual neurons transmit information using chemical and electrical signals, and are organized in groups or circuits involved in different functions.^[^ ^3^, ^5^, ^6^ ^]^ The electrochemical interactions of neuronal ensembles result in electrical activity emerging from the brain, which can show synchrony across populations in the form of brain rhythms and waves that propagate^[^ ^7^, ^8^, ^9^ ^]^ or asynchronous discharges, depending on the brain state.^[^ ^10^ ^]^ Different brain states (slow wave sleep, REM, wakefulness, anesthesia, etc.) are associated with specific network activity parameters such as brain rhythms of certain frequency and synchronization across areas or particular functional connectivity patterns, while brain states are also associated with specific behaviors.^[^ ^1^, ^11^ ^]^ Transitions across brain states are therefore linked to substantial changes in brain activity patterns.^[^ ^12^ ^]^ For example, deep sleep is dominated by large and synchronous brain waves, which transition towards a more desynchronized and higher‐frequency content with waking up.^[^ ^8^, ^13^, ^14^ ^]^ These changes in brain and behavioral states and the concomitant changes in electrophysiological activity are physiologically driven largely by neuromodulators, of which acetylcholine (ACh) is one.^[^ ^12^, ^15^ ^]^ Indeed, it is known that the activation of cholinergic nuclei in the upper brainstem contributes to the transition from sleep to awake states^[^ ^16^, ^17^, ^18^ ^]^ and associated brain patterns.^[^ ^19^ ^]^ However, it is not fully understood how the different cells expressing ACh receptors contribute to the alteration of the global cortical state, or what the contribution of different muscarinic receptor subtypes to this transition is.

Cholinergic receptors include ionotropic nicotinic ion channels and muscarinic metabotropic G protein‐coupled receptors. They modulate brain activity, and in particular the activity of the cerebral cortex,^[^ ^20^ ^]^ being involved in crucial neocortical functions such as attention,^[^ ^21^, ^22^, ^23^ ^]^ learning,^[^ ^24^, ^25^, ^26^ ^]^ memory,^[^ ^27^ ^]^ as well as sensory and motor functions.^[^ ^28^, ^29^, ^30^ ^]^ In the neocortex, ACh is released mostly at cholinergic terminals from neurons with somas in the basal forebrain nuclei. Electrical stimulation of the nucleus basalis can evoke the release of ACh in the neocortex, although in an unselective manner, as ascending projections from basal forebrain nuclei not only comprise cholinergic axons, but also GABAergic and glutamatergic axons.^[^ ^31^ ^]^

Selective stimulation of cholinergic projections in the neocortex from basal forebrain nuclei has been demonstrated with optogenetics and has enabled the disruption of neocortical synchronous activity during certain sleep states.^[^ ^13^ ^]^ Optogenetic stimulation of basal forebrain cholinergic neurons also revealed their influence in awake cortical dynamics: cholinergic neuromodulation was found to be relevant for visual discrimination tasks, modulating the encoding properties of V1 neurons and activating cortical transitions faster than previously presumed.^[^ ^13^ ^]^ However, optogenetics cannot target endogenous signaling as it is limited by the overexpression of microbial proteins using genetic manipulation, which can distort synaptic physiology.^[^ ^32^, ^33^ ^]^ It also raises safety and regulatory concerns regarding therapeutic applications.^[^ ^32^ ^]^ Illumination can be applied locally with implanted devices like optical fibers and LEDs, or transcranially when using long wavelengths, which are less scattering and more penetrating in tissue.^[^ ^34^, ^35^ ^]^

The control of neuronal signaling with photopharmacology is based on synthetic ligands that target endogenous proteins, and thus its physiological relevance spans from circuit to sub‐cellular levels. Since neuronal receptors are highly conserved, photoswitchable ligands can generally be used in multiple species, and their safety and regulation can be established in the same manner as other drugs. Given that the cholinergic system is key to the modulation of a variety of Central Nervous System (CNS) functions,^[^ ^20^, ^36^ ^]^ the use of selective and photoswitchable cholinergic drugs to achieve a spatiotemporal modulation of cortical activity might have relevant scientific and clinical implications. We recently developed a photoswitchable agonist of M2 muscarinic acetylcholine receptors (mAChRs), Phthalimide‐Azo‐Iper (PAI),^[^ ^37^ ^]^ but its action on neuronal circuits and networks has never been explored.

Herein, we report that the cholinergic‐dependent brain state transitions in the neocortex can be controlled and thus investigated in detail with light using photosensitive drugs. In particular, we have found that PAI enables the modulation of spontaneous emerging slow oscillations (SO) in neuronal circuits. PAI *cis*‐to‐*trans* photoisomerization decreases the Down state durations and increases oscillatory frequency in cortical slices. In addition, PAI allows the reversible manipulation of the cortical oscillatory frequency in anesthetized mice using light. Two different species, ferret (in vitro) and mouse (in vivo) were used in this study, since ferret cortical slices are more suitable than those of rodents to spontaneously generating network activity under physiological conditions.^[^ ^38^ ^]^ Thus, we demonstrate that photopharmacology allows us to selectively control SO both in vitro and in vivo, opening the way for the analysis of their spatiotemporal dynamics and their effects on brain and behavioral state transitions. Photoactivation of mAChR driven by PAI offers the potential to modulate neural circuits in numerous regions distributed throughout the neocortex. In this first work we studied the impact of global activation on the temporal dynamics of cortical patterns. Patterned illumination will allow us to carry out more spatially targeted studies in the future.

In our design, we considered that changes in cortical rhythms accompany behavioral state transitions, and endogenous ACh actions play a central role in such variations.^[^ ^11^, ^39^, ^40^, ^41^, ^42^, ^43^, ^44^, ^45^, ^46^ ^]^ It is known^[^ ^16^, ^17^, ^18^ ^]^ that ACh contributes to the shift of the neocortical network state from synchronous to asynchronous activity (associated with awake states), thus we hypothesized that a preparation expressing synchronized activity—physiological slow oscillations—would be an appropriate testbed to investigate the effect of muscarinic ligands, including the novel photosensitive agonist PAI.

## 2 Results

### 2.1 Non‐Specific Activation of mAChRs Evokes Neuronal Hyperexcitability in Cortical Slices

We started by studying the effect of Iperoxo, a muscarinic non‐selective agonist,^[^ ^47^, ^48^ ^]^ on cortical SO spontaneously generated in cortical slices. The goal was to evaluate the potential of mAChRs to modulate the dynamics of the neocortical network, while avoiding the simultaneous activation of nicotinic cholinergic receptors (nAChRs).^[^ ^20^ ^]^ In addition, these experiments would allow the validation of whether Iperoxo‐based photoswitches like PAI could be useful photopharmacological tools to control neuronal activity, and characterizing their performance.

**Figure 1:**
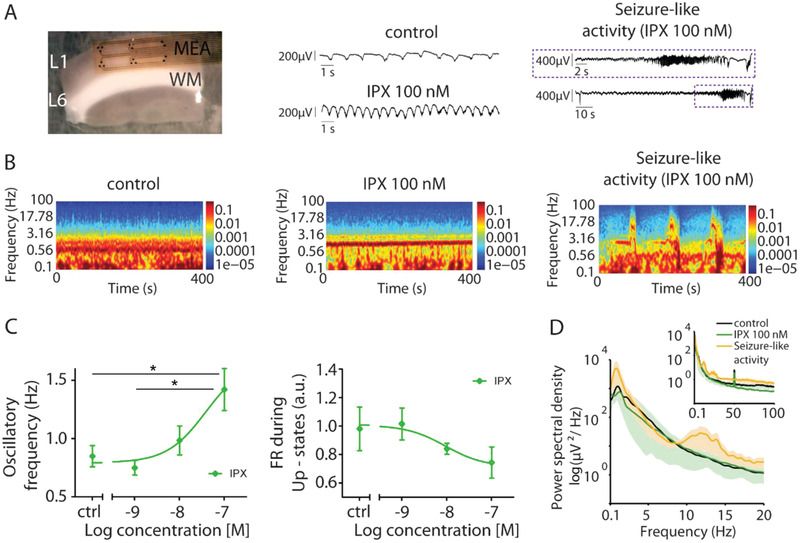
Activation of mAChRs with non subtype‐specific agonist Iperoxo (IPX) evokes neuronal hyperexcitability in cortical slices. A) On the left, the experimental setup: 16‐channel multielectrode array (MEA); WM, white matter; L1‐L6, layer 1–6. On the right, raw local field potential (LFP) traces illustrating network activity showing the increase in oscillatory frequency corresponding to the spectrogram of panel B with 100  × 10^–9^ m IPX. B) Spectrogram from the same time recording of LFP traces on panel A: control, 100  × 10^–9^ m and periods of seizure‐like discharges. C) Oscillatory frequency (Hz) and FR (a.u.) during the Up‐states in control (ctrl) conditions and after IPX (1, 10, 100  × 10^–9^ m; *n =* 7 ferret brain slices). Muscarinic activation of the brain slice with 100  × 10^–9^ m IPX produces a significant increase in oscillatory frequency compared to control conditions, and no significant changes in the firing rate (FR) during Up‐states. Data are reported as mean ± standard error of mean (SEM). Analyses were performed with one‐way ANOVA test (Brown–Forsythe and Welch test, unpaired t with Welch's correction). *p‐value *<*5 × 10^−2^. D) Averaged power spectral density (PSD) of oscillatory activity showing low (<1 Hz), delta (1–4 Hz) and alpha (7–12 Hz) frequency component.

Ferret cortical slices spontaneously generate cortical SO,^[^ ^38^ ^]^ a hallmark of activity during deep sleep or anesthesia.^[^ ^38^ ^]^ We recorded this spontaneous oscillatory activity (control) and then the activity under different concentrations (1, 10, 100  × 10^–9^ m; *n =* 7) of Iperoxo. Slow oscillations consist of alternating periods of activity or high neuronal firing (Up states) and periods of near silence (Down states). In order to quantify Up and Down state duration, firing rates and frequencies, we estimated the MUA (multiunit activity reflecting the activity of the local neuronal population) and ran an algorithmic separation of Up and Down states by establishing a threshold from a bimodal distribution of MUA that separates firing periods (Up states) from silent periods (Down states).^[^ ^7^ ^]^ A more detailed account of this quantification can be found in the Experimental Section. The activation of mAChRs by Iperoxo resulted in a global change in the network's dynamics in a dose‐dependent way, displaying enhanced excitability (**Figure** **1**A,B). At 100  × 10^–9^ m Iperoxo, the oscillatory frequency—or frequency of the Up/Down state cycle—significantly increased (from 0.85 ± 0.1 Hz in the control to 1.42 ± 0.18 Hz with 100  × 10^–9^ m Iperoxo, *p*‐value = 3 × 10^−2^), while the firing rate (FR; see Experimental Section)^[^ ^49^ ^]^ during Up‐states did not change significantly (from 0.97 ± 0.15 a.u. to 0.74 ± 0.09 a.u. with 100  × 10^–9^ m Iperoxo) (Figure 1C). At concentrations equal or higher than 100  × 10^–9^ m Iperoxo, the oscillatory activity evolved to periods of full‐blown epileptiform discharges (Figure 1A,B), as previously characterized in this preparation^[^ ^49^ ^]^ and also described in vivo following muscarinic activation,^[^ ^50^, ^51^ ^]^ as in the pilocarpine model of epilepsy.^[^ ^52^ ^]^ Such epileptiform or seizure‐like discharges are a clear sign of hyperexcitability, displaying intense firing for several seconds and long periods of silence, and characterized by a rather different spectrogram showing larger amplitudes in different frequency bands: slow (<1 Hz), delta (1–4 Hz), and alpha (7–12 Hz) frequency components, and including enhancement of beta (12–30 Hz) and gamma frequencies (30–100 Hz) (Figure 1D). However, our interest was to avoid epileptiform discharges and to activate muscarinic receptors within the range of physiological activity, therefore with the activation achieved below 100  × 10^–9^ m Iperoxo.

### 2.2 Effect of PAI Isomers on Slow and Fast Oscillations In Vitro

**Figure 2:**
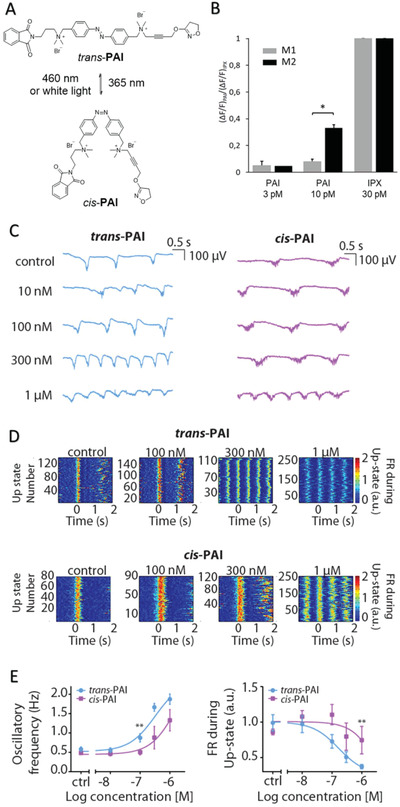
Effect on SO of mAChRs activation by *trans*‐PAI (dark‐relaxed) and *cis*‐PAI (pre‐illuminated with UV). A) Chemical structures of *trans*‐ and *cis*‐PAI. B) Subtype selectivity of *trans*‐PAI (3, 10 × 10^–12^ m) was studied by comparing the amplitude of fluorescence calcium imaging responses of cells expressing M1 (3 × 10^–12^ m, *n =* 232 cells; 10 × 10^–12^ m, *n =* 232 cells; IPX, *n =* 258) or M2‐GqTOP (3 × 10^–12^ m, *n =* 70 cells; 10 × 10^–12^ m, *n =* 200 cells; IPX, *n =* 300) mAChRs, using the procedure described in Riefolo et al. 2019^[^ ^37^ ^]^ and by pre‐incubating cells with OGB‐1AM (10 × 10^–6^ m for 30 min) as calcium indicator. M2 mAChR transfected cells gave a significantly higher response than M1 mAChR expressing cells (33% for M2 compared to 8% for M1). Data were normalized over the maximum response obtained with the nonselective orthosteric agonist IPX in saturation conditions at 30 × 10^–12^ m. These results in cell lines are in agreement with previous reports (Riefolo et al., 2019),^[^ ^37^ ^]^ in which higher concentrations were required for cardiac assays in vivo. Bars are mean ± SEM and compared using a *t*‐test of two samples assuming equal variances. *p < 5 × 10^−3^. C) Raw local field potential (LFP) example recordings showing the different ability of *trans*‐ and *cis*‐PAI to increasing the oscillatory frequency. Note that *trans*‐PAI is a stronger agonist of M2 mAChR than *cis*‐PAI.^[^ ^37^ ^]^ D) Raster plots showing the FR during the Up‐states (color coded) under control conditions and different *trans*‐ and *cis*‐PAI concentrations. E) oscillatory frequency (Hz) and FR during the Up‐states (a.u.) of the two different PAI isomers, *trans*‐ (blue, *n* = 6 ferret brain slices) and *cis*‐PAI (pink, *n* = 6 ferret brain slices) at different concentrations. Data are reported as mean ± SEM. Analyses were performed with multiple *t*‐test (Mann‐Whitney). Significant differences between *cis*‐ and *trans*‐PAI (**p‐value < 10^−2^) are observed in the oscillatory frequency at 100  × 10^–9^ m and in the FR at 1  × 10^–6^ m. These experiments are aimed at estimating the concentration of effective photoswitching of cortical oscillations, which was set at 200  × 10^–9^ m in subsequent experiments (see Figures 3 and 4).

The hyperexcitable network state induced with Iperoxo reflects the impact of mAChR activation on cortical networks and brain states.^[^ ^14^ ^]^ In order to remotely control these states, we aimed at the muscarinic neuromodulation with light using PAI, a photoswitchable Iperoxo‐derivative that allows the reversible activation of M2 mAChRs in vivo.^[^ ^37^ ^]^ The light‐dependent behavior of PAI is achieved with a molecular switch in its structure that is based on azobenzene. PAI exists in two forms, *trans* and *cis*, which are in dynamic equilibrium with each other (**Figure** **2**A). A distribution of 87% in favor of the *trans* form (13% of the *cis*) is found in the dark or after illumination with visible light (white light (WL) for 2 min). After illumination with ultraviolet (UV) light (365 nm for 1 min) the ratio between the two configurations rapidly changes to about 77% *cis* (23% *trans*). Both PAI isomeric mixtures (respectively termed “*trans*” and “*cis*” for simplicity) are thermally stable for hours and their biological effects have been characterized in vitro and in vivo.^[^ ^37^ ^]^ Despite the partial photoconversion (which is characteristic of azobenzene‐based switches) the *trans*‐ and the *cis*‐enriched forms of PAI display different pharmacological activity: the *trans* configuration is a more potent activator of M2 receptors than the *cis*, allowing the reversible manipulation of muscarinic functions with light over many cycles.^[^ ^37^ ^]^ Prior to testing PAI in the brain, which shows high expression of both M1 and M2 receptors, we tested its activity profile in these two mAChR subtypes using in vitro GTPgammaS assays (Figure S1.2, Supporting Information) and calcium imaging assays (Figure 2B) in cultured cell lines separately overexpressing M1 and M2 receptors. Our results show significant M2 selectivity (Figure 2B) in agreement with the dualsteric design and in vivo properties of PAI.^[^ ^37^ ^]^ M2 receptors are highly expressed in cardiac tissue and in the brain, where they play a relevant role in several CNS disorders.^[^ ^36^ ^]^ Controlled spatiotemporal regulation of M2 mAChR activity and subsequent effects on cortical neuronal networks may provide new therapeutic opportunities for diseases involving the cholinergic system. Note that subtype selectivity can be advantageous to interpret (photo)pharmacological experiments but muscarinic activation with light is sufficient for the aim of this work. We applied PAI to spontaneously active neocortical brain slices and recorded their oscillatory activity before and after photoactivating the drug. We first obtained dose‐response curves of the two drug forms separately, *trans*‐ (dark‐adapted state) and *cis*‐PAI (pre‐illuminated with UV light) with the purpose of identifying the concentration range displaying differences in brain activity with light. The baseline activity (characterized by SO) was recorded as a control, prior to bath‐application of artificial cerebrospinal fluid (ACSF) with increasing PAI concentrations (10  × 10^–9^ m, 100  × 10^–9^ m, 300  × 10^–9^ m, and 1  × 10^–6^ m, *n* = 6 ferret brain slices for each PAI form, *trans* and *cis*) (Figure 2C). Both *trans*‐PAI and *cis‐*PAI significantly modulated the Up‐ and Down‐state sequence in a dose‐dependent manner, with *cis‐*PAI displaying weaker effects in agreement with the reported PAI properties in cells expressing M2 receptors^[^ ^37^ ^]^ (Figure 2C,D,E; the quantification and significance versus control for each isomer are detailed in Figure S2, Supporting Information). The differences between *cis‐*PAI and *trans*‐PAI were significant for the oscillatory frequency at 100  × 10^–9^ m and for the FR during Up‐states at 1  × 10^–6^ m (Figure 2E). Note that epileptiform activity was never observed with PAI up to concentrations of 1 × 10^–6^ m, in contrast to the effect of Iperoxo.

These results obtained with a limited number of animals for each isomer (*n =* 6) already suggested that intermediate concentrations between 100 and 300  × 10^–9^ m might be suitable to produce changes in the oscillatory frequency and in the firing rate of the Up‐states upon *cis‐trans* photoisomerization (Figure 2E). Thus, in subsequent experiments with light we expanded the sample (*n =* 17) and focused on the concentration of 200  × 10^–9^ m in order to photomodulate cortical SO using PAI.

### 2.3 Photoswitchable PAI Effectively Modulates Cortical Slow Oscillations In Vitro

**Figure 3:**
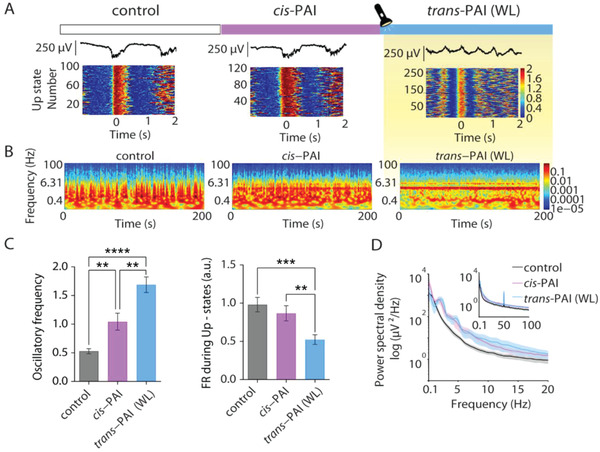
Photocontrol of brain waves in vitro using PAI and direct illumination with white light. A) Representative local field potential (LFP) traces (top) and raster plots of firing rate (FR) during the Up‐states under control conditions, 200  × 10^–9^ m of *cis*‐PAI and 200  × 10^–9^ m *trans*‐PAI after photoconversion with white light (WL) (*n* = 17 ferret slices) (bottom). B) Representative spectrogram under control condition, 200  × 10^–9^ m of *cis*‐PAI and 200  × 10^–9^ m *trans*‐PAI (WL). C) Oscillatory frequency (Hz) and FR during the Up‐states (a.u.) at 200  × 10^–9^ m PAI after pre‐illumination with 365 nm (*cis*‐PAI), and photoswitching with WL (*trans*‐PAI). Data are reported as mean ± SEM. Analyses were performed with one‐way ANOVA test (Brown–Forsythe and Welch test, unpaired *t* with Welch's correction). **p‐value < 10^–2^; ***p‐value < 10^–3^, ****p‐value < 10^–4^. D) Averaged power spectral density (PSD) of oscillatory activity under control conditions, 200  × 10^–9^ m of *cis*‐PAI and 200  × 10^–9^ m *trans*‐PAI after WL activation (color code).

Once the concentration range of drug to obtain different oscillatory activity evoked by *cis‐* and *trans*‐PAI was determined in vitro (Figure 2), we moved on to control the rhythmic activity with light in cortical slices (**Figure** **3**). We took advantage of the thermal stability of both PAI forms to apply initially the less potent one (*cis*‐PAI) at 200  × 10^–9^ m in cortical slices (*n* = 17), in the absence of WL to avoid photoconversion to *trans*‐PAI during the recordings.^[^ ^37^ ^]^ As shown in Figure 3, 200  × 10^–9^ m *cis*‐PAI evoked an increment of the oscillatory frequency (from 0.53 ± 0.04 Hz in the control to 1.04 ± 0.14 Hz with *cis*‐PAI, *p*‐value = 2.8 × 10^−3^), and no significant effects in the FR of the Up‐states (from 0.98 ± 0.09 a.u. in the control to 0.86 ± 0.10 a.u. with *cis*‐PAI, *p*‐value = 2.9 × 10^−1^) in comparison to the control situation (Figure 3A,C). Subsequent direct illumination of the slices with WL produced a robust increase in oscillatory frequency (from 0.53 ± 0.04 Hz in the control to 1.68 ± 0.13 upon illumination, *p‐*value < 10^−4^; from 1.04 ± 0.14 Hz with *cis*‐PAI to 1.68 ± 0.13 upon illumination, *p*‐value = 1.1 × 10^−3^), a significant decrease in FR of the Up‐states (from control values of 0.98 ± 0.09 a.u. to 0.52 ± 0.06 a.u. upon illumination, *p*‐value = 2 × 10^−4^; from 0.86 ± 0.10 a.u. with *cis*‐PAI to 0.52 ± 0.06 a.u. upon illumination, *p*‐value = 2.2 × 10^−3^), and a noticeable change in the activity regime of the network (Figure 3A,C). The changes in the power spectrum in the population were incremental, as illustrated in Figure 3B,D. These differences are in agreement with PAI photoconversion to the active form (*trans*). At this stage, the modulation of cortical activity was not reversible upon illumination with 365 nm light (to isomerize PAI to the *cis* form in situ) in slices, which could be due either to pharmacological irreversibility (which was ruled out in experiments in vivo, see next Section 2.4) or to the reduced penetration of UV light in brain tissue.

### 2.4 Photoswitchable PAI Can Reversibly Modulate Brain Wave Activity In Vivo

**Figure 4:**
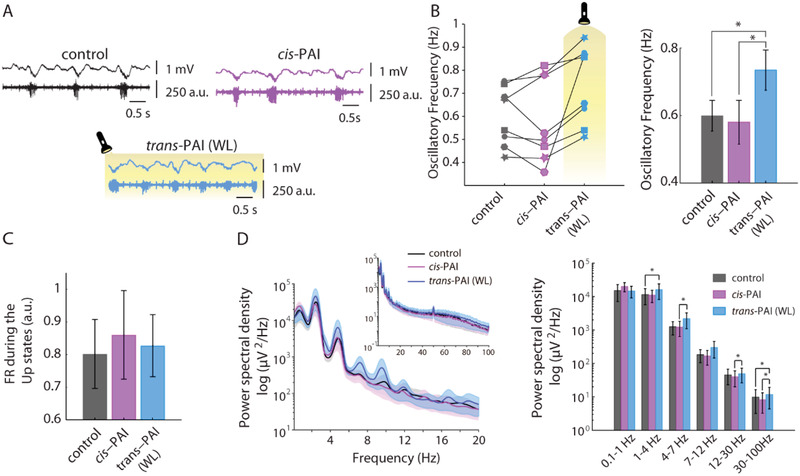
In vivo photomodulation of brain waves. A) Representative raw traces of local field potential (LFP) (top, in mV) and multiunit activity (bottom, in arbitrary units), showing the differences in oscillatory frequency and firing rate (FR) during the Up‐states between the control, 1 x 10^–6^ m *cis*‐PAI (pre‐illuminated with 365 nm), and *trans*‐PAI after photoswitching with white light (WL). B) Individual (left) and mean (right) quantification of oscillatory frequency (Hz) (*n =* 8 mice), showing a significant increase upon illuminating *cis*‐PAI with WL. Data are reported as mean ± SEM. Analyses were performed with one‐way ANOVA test (repeated measures [RM], Geisser–Greenhouse correction—no sphericity—and uncorrected Fisher's LSD). **p‐value < 10^–2^; ***p‐value < 10^–3^, ****p‐value < 10^–4^. *p‐value < 5 × 10^−2^. C) The mean quantification of FR during the Up‐states (a.u.) is not significantly affected by illumination of *cis‐*PAI (*n =* 8). D) Averaged power spectral density (PSD) of oscillatory activity at different concentrations displays significant enhancement at delta, theta, alpha, and gamma frequency bands after WL activation (left). Mean ± SEM quantification of PSD from D‐left panel (*n =* 8) (right). Analyses were performed with Friedman test and the Wilcoxon post‐hoc tests corrected for multiple comparisons. *p‐value < 5 × 10^−2^.

Having established the unique ability of PAI to alter cortical oscillatory activity with light in slices, we then aimed at obtaining a proof of concept of photocontrolling the cortical state in vivo. Cortical activity was recorded from C57BL6/JR mice (*n* = 8) with an electrode inserted through a craniotomy across which we used to carry out the drug application and brain illumination (see Experimental Section). Initially, we induced deep anesthesia in the animals, a state that is known to reproduce the slow wave sleep state,^[^ ^7^, ^41^, ^53^ ^]^ and which is characterized by the generation of cortical SO similar to the slow frequency waves observed in our experiments in slices under control conditions (Figures 1, 2, 3).^[^ ^7^ ^]^ Such SO activity in anesthetized mice was recorded for 500 s under WL illumination of the brain, and the characteristic parameters obtained (oscillatory frequency 0.60 ± 0.05 Hz, FR during the Up‐states 0.80 ± 0.11 a.u.) were taken as the baseline, control condition in vivo. A 100 µL drop of 1 × 10^–6^ m *cis*‐PAI solution was initially applied to the brain surface in the absence of WL, to avoid *cis* to *trans* photoisomerization of PAI. The oscillatory frequency was not significantly altered by *cis*‐PAI (0.58 ± 0.06 Hz) (**Figure** **4**B), while it caused only a minor increase in the FR during the Up‐states (0.86 ± 0.14 a.u.) (Figure 4C). Subsequent illumination with WL significantly increased the oscillatory frequency (from 0.60 ± 0.05 Hz in the control to 0.73 ± 0.06 Hz upon illumination, *p* = 2.8 × 10^−2^) (Figure 4B), and induced an increment of the delta (*p* = 1.6 × 10^−2^) and gamma (*p* = 2.3 × 10^−2^) frequencies band compared with control conditions (Figure 4D), while the FR during Up states was decreased to the control values (0.83 ± 0.09 a.u., Figure 4C). This light‐dependent increase in oscillatory frequency activity of PAI in vivo is in partial agreement with the in vitro results observed in ferret slices, which also displayed a reduction in FR (Figure 3). The difference may be due to the integrity of the neural network and the cholinergic activity in vivo.

In brain cortical sections the effect of *trans*‐PAI is not reversible using 365 nm light and we aimed to understand whether this was due to muscarinic activation mechanisms or just to the limited tissue penetration of UV wavelengths, which could hinder isomerization in deeper regions. The illumination of the mice brain using a more powerful 365 nm source (100 W bulb, see Experimental Section) did not significantly reduce the oscillatory frequency from the *trans*‐PAI‐excited situation (*n =* 4, Figure S4A, Supporting Information). Since tissue scattering cannot be avoided at this wavelength we tried to revert the effect of *trans*‐PAI by outcompeting it by direct application of its *cis* isomer, which binds to the same receptor site with similar strength that *trans* (see competition binding experiments, Figure S1.1, Supporting Information) but causes weaker or no activation. For this purpose, we significantly increased the oscillatory frequency with 200  × 10^–9^ m *trans*‐PAI, and then applied 1 × 10^–6^ m *cis*‐PAI. Indeed, we observed the return to the control‐like oscillatory activity (*n =* 4, Figure S4BC, Supporting Information), demonstrating that the process is pharmacologically reversible and that the lack of reversibility is probably due to the limited penetration of UV light. Thus, the optimization of both the light delivery system and/or drug photophysical properties (e.g., photoswitching at less scattering wavelengths) have the potential to reversibly photocontrol neural muscarinic actions in vivo.

## 3 Discussion

Different brain states are associated with distinct brain emergent patterns and behaviors. In order to establish strong causal links between them, neural activity must be manipulated and recorded to observe the effects on specific behaviors. Pharmacological tools have proven very useful but they affect brain waves in a systemic way and have relatively slow dynamics.^[^ ^54^ ^]^ Thus, understanding the mechanisms of brain and behavioral state transitions requires new techniques to manipulate neuronal activity^[^ ^1^ ^]^ that enable the neural modulation of specific brain regions and neuronal circuits in a fast and reversible way. They can be defined by a multimodal stimulation approach (e.g., electrical stimulation with implanted microelectrodes), photostimulation with cell‐specific optogenetics^[^ ^55^, ^56^, ^57^, ^58^ ^]^ and with neurotransmitter‐specific photopharmacology.^[^ ^59^ ^]^

Electromagnetic stimulation pioneered the noninvasive modulation of brain activity and is used therapeutically to treat CNS diseases.^[^ ^60^ ^]^ For example, transcranial alternating current stimulation has been applied to modulate alpha and beta waves in the motor cortex^[^ ^61^ ^]^ and transcranial magnetic stimulation has been used to modulate gamma oscillations in the prefrontal cortex.^[^ ^62^ ^]^ However, further improvements are required to enhance their spatiotemporal and spectral performance, both for fundamental research and therapeutic purposes.^[^ ^60^ ^]^ Optogenetics^[^ ^55^, ^56^, ^57^, ^58^ ^]^ has emerged as an alternative to electromagnetic stimulation, allowing the activation or inhibition of specific cell populations. For example, photocontrolling the release of ACh, which modulates the transitions between different brain states,^[^ ^12^, ^15^ ^]^ can be achieved by overexpressing photosensitive proteins in cholinergic neurons of mice neocortex.^[^ ^13^, ^63^ ^]^ However, genetic manipulation is required in this approach, limiting so far its usability in humans.^[^ ^32^ ^]^ The photopharmacological approach presented here is, to date, the only way to directly photomodulate brain state transitions in intact tissue. We first studied the effect of the muscarinic agonist Iperoxo^[^ ^47^ ^]^ on isolated cortical slices (Figure 1) in order to demonstrate that a slow oscillatory state can be controlled by selectively manipulating muscarinic receptors (including all M1–M5 subtypes) at their physiological location and context. The oscillatory frequency of the network was increased with 100  × 10^–9^ m Iperoxo, eventually leading to seizure‐like discharges, in agreement with the outcome of muscarinic stimulation using knockout mice and the pilocarpine‐induced model of epilepsy.^[^ ^50^, ^51^ ^]^ Photocontrol of muscarinic signaling was subsequently achieved in vitro and in vivo with the photochromic Iperoxo derivative PAI^[^ ^37^ ^]^ (Figures 2, 3, 4). We showed that PAI is selective for M2 versus M1 subtype receptors and thus it only accounts for part of the effects of Iperoxo. In the experiments of Figure 2, PAI was pre‐illuminated in order to administer the inactive *cis* form, which is then stable over hours at 37 °C. This allowed us to compare the activities of both forms at different concentrations and identifying 200  × 10^–9^ m PAI as a suitable concentration for direct photoswitching of cortical activity in vitro (Figure 3) and in vivo (Figure 4). Overall, this proof‐of‐concept demonstration of photocontrol of brain waves has allowed us to identify two important limitations of PAI that will be addressed in future drug designs. Namely, that PAI is active in the dark and that it is not readily photo‐reversible in brain tissue due to the limited penetration of UV light. We have promising evidence that both limitations can be overcome, respectively using a “bridged” azobenzene to obtain a dark‐inactive analog drug with equivalent pharmacology,^[^ ^64^ ^]^ and using tissue‐penetrating infrared light from a pulsed laser.^[^ ^37^ ^]^ Other aspects of PAI that can be optimized include its activation wavelength under non‐pulsed illumination, photosensitivity, reversibility, photoswitchable concentration range (“therapeutic window”), safety profile, and permeability to the blood‐brain barrier. Still, as a small molecule, PAI is less likely to trigger adverse immune responses than the overexpression of microbial opsins.^[^ ^32^, ^65^ ^]^ M2 mAChRs are involved in several CNS diseases like major depressive^[^ ^36^, ^66^ ^]^ and bipolar disorders,^[^ ^36^, ^67^ ^]^ Parkinson's^[^ ^36^, ^68^ ^]^ and Alzheimer's^[^ ^36^, ^68^ ^]^ diseases, but also in alcohol, smoking, and drug dependence.^[^ ^36^, ^69^ ^]^ These disorders are thus susceptible to drug‐based photomodulation in vivo without requiring genetic manipulation. We envisage that once a suitable drug concentration has been determined for optimal photoswitching, spatiotemporal patterns of stimulation can be applied to ask specific questions. For example, how does a focal, acute activation propagate in space and time? What is the behavioral outcome? How does it interfere with the ongoing (endogenous) wave activity? How does the size, duration, and location of the activated region relate to the behavioral outcome and its reversibility? Beyond the fundamental understanding of the mechanisms of brain waves, if these experiments could be performed with safe photoswitchable drugs and using noninvasive transcranial illumination, these questions would gain great clinical relevance and feasibility. Beyond photoswitchable muscarinic agonists, these concepts could be also exploited with drug‐like antagonists (Riefolo et al, in review) and adrenergic ligands^[^ ^70^ ^]^ among many others, eventually allowing us to build 3D functional pharmacological charts that could be correlated with anatomical maps.

In summary, the manipulation of brain state transitions, by means of photocontrolling the frequency of cortical oscillations, has been achieved with a photoswitchable dualsteric agonist of M2 mAChRs. This result opens the way to: a) dissecting the spatiotemporal distribution and pharmacology of brain states, namely mapping how they depend on agonists, antagonists, and modulators of the different muscarinic subtypes expressed in the CNS; and b) investigating the neuronal dynamics and causality that regulate brain state transitions in the cerebral cortex and beyond. In particular, two‐photon stimulation of PAI using pulsed infrared light^[^ ^37^ ^]^ should enable deep penetration and subcellular resolution in 3D,^[^ ^71^ ^]^ as recently demonstrated with endogenous mGlu_5._^[^ ^72^ ^]^ Compared to the local and often inhomogeneous expression patterns achieved with viral injections of optogenetic constructs, diffusible small molecules like PAI can in principle be applied to larger brain regions to control neuronal oscillations.^[^ ^73^ ^]^ Thus, remote control of brain waves based on the photopharmacological manipulation of endogenous muscarinic receptors may reveal the complex 3D molecular signaling underlying brain states and their transitions, in order to link them with cognition and behavior in a diversity of wild‐type organisms.

## 4 Conclusion

A method for directly manipulating neural activity and brain rhythms with light‐controlled drugs is demonstrated. It is based on a photoswitchable muscarinic small molecule and does not require gene therapy. The photocontrol of endogenous receptors and their functions in the central nervous system, such as the transition between different brain states, is an achievement for neuromodulation technologies that is useful as a tool in basic neuroscience research and in future brain therapies and stimulation. Thus, photopharmacological neuromodulation combined with implantable optoelectronic devices offers the ability to exploit the untapped potential of neuropharmacology, by controlling drug action in precise spatiotemporal patterns.

## 5 Experimental Section

### Slice Preparation

For the in vitro experiments the authors used isolated cortical slices from ferret, because they robustly reproduce the cortical SO compared with other animal models.^[^ ^74^ ^]^ Twenty‐seven ferrets (4‐ to 6‐months‐old) were anesthetized with sodium pentobarbital (40 mg kg^−1^) and decapitated. The entire forebrain was rapidly removed and placed in oxygenated cold (4–10 °C) bathing medium.^[^ ^75^ ^]^ Ferrets were treated in accordance with protocols approved by the Animal Ethics Committee of the University of Barcelona, which comply with the European Union guidelines on the protection of vertebrates used for experimentation (Directive 2010/63/EU of the European Parliament and the Council of 22 September 2010). Coronal slices (400 µm thick) from primary visual cortex (V1) were used.^[^ ^76^ ^]^ To increase tissue viability the authors used a modification of the sucrose‐substitution technique,^[^ ^77^ ^]^ such that during slice preparation, the tissue was placed in a solution in which NaCl was replaced with sucrose while maintaining the same osmolarity. After preparation, the slices were placed in an interface‐style recording chamber (Fine Sciences Tools, Foster City, CA, USA). During the first 30 min the cortical slices were superfused with an equal mixture in volume of the normal bathing medium, artificial cerebral spinal fluid (ACSF), and the sucrose‐substituted solution. Following this, normal bathing medium was added up to the recording chamber and the slices were superfused for 1–2 h; the normal bathing medium contained (in mm): NaCl, 126; KCl, 2.5; MgSO_4_, 2; Na_2_HPO_4_, 1; CaCl_2_, 2; NaHCO_3_, 26; dextrose, 10; and was aerated with 95% O_2_, 5% CO_2_ to a final pH of 7.4. Then, a modified slice solution was used throughout the rest of the experiment; it had the same ionic composition except for different levels of the following (in mm): KCl, 4; MgSO_4_, 1; and CaCl_2_, 1.^[^ ^75^ ^]^ Bath temperature was maintained at 34–36 °C.

### Drug Application and Photostimulation in Brain Slices

Iperoxo and PAI, both prepared as previously reported from commercially available starting materials^[^ ^37^ ^]^ were bath‐applied at the concentrations range of 1 to 100  × 10^–9^ m for Iperoxo and 10  × 10^–9^ m to 1 × 10^–6^ m for PAI, as discussed in the Results section. The authors typically waited more than 1000 s after the application of the drug in order to let it act, to obtain a stable pattern of electrical activity, and to ensure a stable concentration in the bath, this being an interface chamber. PAI effectively photomodulates the activity of M2 receptors in vitro and in vivo: its dark‐adapted state (*trans* form) behaves as a strong M2 agonist, then upon illumination with UV light (365 nm), PAI switches to its less active state (*cis* form). PAI can be switched back to its “full on‐state” with WL, or using two‐photon excitation with pulsed near‐infrared light.^[^ ^37^ ^]^ The high thermal stability of PAI *cis* form allows the administration of the less active drug (inactive in the cerebral cortex at concentrations lower than 1 × 10^–6^ m) and subsequent activation of M2 receptors in the target region with WL.^[^ ^37^ ^]^ The authors first investigated the efficacy of PAI in cortical neuronal circuits in vitro by obtaining the dose‐response curves of *trans*‐ and *cis*‐PAI solutions applied separately. The more active PAI isomer (*trans*) was tested by applying its dark‐adapted form (87% of *trans*‐PAI), and *cis*‐PAI was obtained by illuminating 1 mm stock solutions with 365 nm light (77% of *cis*‐PAI, which is the maximum that can be achieved at the photo stationary state). Because of the high concentration of the stock solution (1 mm) and for the sake of having the maximal possible photoconversion of PAI into the *cis* form, UV light irradiation (Vilber Lourmat Dual Wave Length UV Lamps, 365 nm 6 W) was performed over 10 min, even if it was demonstrated that the maximal percentage of the *cis* can be reached by a shorter time exposure to UV light (2 min).^[^ ^37^ ^]^ Increasing concentrations of both *trans*‐ and *cis*‐PAI (10  × 10^–9^ m, 100  × 10^–9^ m, 300  × 10^–9^ m, and 1  × 10^–6^ m) were bath applied in order to build up the dose‐response curves.

### LFP Recording and Data Analysis from In Vitro Recordings

The authors' objective in this study was to identify the modulation of network dynamics exerted by photoswitchable muscarinic agonists. To this end, they obtained multiple LFP recordings and their correspondent multiunit activity (MUA) in the way described below. No single units were identified since they aimed at capturing the dynamics of the population and not of individual neurons. The recordings started after allowing at least 2 h of recovery of the slices. Extracellular recordings were obtained with flexible arrays of 16‐electrodes arranged in columns as in Figure 1A. The multielectrode array (MEA) covered a large part of the area occupied by a cortical slice.^[^ ^78^ ^]^ It consisted of six groups of electrodes positioned to record electrophysiological activity from superficial and from deep cortical layers (692 µm apart) and from what should correspond to three different cortical columns (1500 µm apart). The unfiltered field potential (raw signal) was acquired at 10 kHz with a Multichannel System Amplifier (MCS, Reutlingen, Germany) and digitized with a 1401 CED acquisition board and Spike2 software (Cambridge Electronic Design, Cambridge, UK). The MUA was estimated from the power of the frequencies between 200 and 1500 Hz in 5 ms windows.^[^ ^7^, ^49^ ^]^ The spectrum in this frequency band is a good estimate of the firing of the neuronal population, since it is proportional to the density of the Fourier components at high frequencies.^[^ ^79^ ^]^ The MUA signal values were logarithmically scaled to compensate for the high fluctuations in the firing of neurons that are very close to the electrode, thus obtaining the logMUA signal (which the authors refer to as firing rate; FR). A bimodal distribution of the MUA the two peaks of the distribution corresponded to the samples of the activity network belonging to the Up and Down state, respectively. Thus, a threshold value separating the two modes of the distribution was set between the two peaks, such that samples belonged to the Up or to the Down states depending on their position with respect to this threshold. After Up and Down state detection, mean Up and Down state durations were obtained.^[^ ^7^ ^]^ The frequency of the SO was the inverse of the duration of the entire Up–Down cycle. The Up state detection necessary was performed by setting a threshold in the log(MUA) time series as previously described to quantify frequency of the SO.^[^ ^7^, ^49^ ^]^ Firing rate (FR) of the Up states was quantified from the transformed log(MUA) signal as mean of absolute value of log(MUA). To study the variability of power spectral densities (PSD) of the local field potential (LFP), the authors used Welch's method with 50% overlapped Hamming window with a resolution of 1 Hz. All off‐line estimates and analyses were implemented in MATLAB (The MathWorks Inc., Natick, MA, USA). All variables in the experimental conditions were compared with the control (no chemical added) condition.

### The In Vivo Preparation

Cortical electrophysiology experiments were carried out in 2–3‐month‐old C57BL6/JR mice (*n* = 8) in accordance with the European Union Directive 2010/63/EU and approved by the local ethics committee. Mice were kept under standard conditions (room temperature, 12:12‐h light‐dark cycle, lights on at 08:00 a.m.). Anesthesia was induced by intraperitoneal injection of ketamine (30 mg kg^−1^) and medetomidine (100 mg kg^−1^). After this procedure, the mouse was placed in a stereotaxic frame, and air was enriched with oxygen. Body temperature was maintained at 37 °C throughout the experiment.^[^ ^7^ ^]^ A craniotomy was performed in each mouse: AP −2.5 mm, L 1.5 mm (primary visual cortex, V1).^[^ ^80^ ^]^ Cortical recordings were obtained from infragranular layers with 1–2 MΩ single tungsten electrode insulated with a plastic coating except for the tip (FHC, Bowdoin, ME, USA). Spontaneous LFP recordings from the V1 area provided information about the local neuronal population activity—within 250 µm.^[^ ^81^ ^]^ MUA estimation, Up‐state detection and quantification of relative FR was performed as previously described. All these parameters were used to compare spontaneous activity during anesthesia (control), after application of the pre‐illuminated less active drug form (*cis*‐PAI) and drug activation with WL (*trans*‐PAI). *Cis*‐PAI was locally delivered to the cerebral cortex surface and activity was recorded while applying a commercial red filter on the WL source to avoid the activation of the drug. The uncovered brain was illuminated from a 3 cm distance with a WL source (Photonic Optics Optics Cold Light Source LED F1) in order to activate the drug in situ (*trans*‐PAI). The *trans*‐to‐*cis* photoconversion was performed by illuminating the brain from a 5 cm distance with a very powerful 365 nm lamp (Spectroline, Spectronics FC‐100‐F, 230 V, 365 nm, 100 W Spot Bulb) in order to improve the penetration of light into the tissues. The electrophysiological signal was amplified with a multichannel system (Multi Channel Systems), digitized at 20 kHz with a CED acquisition board, and acquired with Spike 2 software (Cambridge Electronic Design) unfiltered.^[^ ^82^ ^]^

### In Vitro Specific M1 and M2 Muscarinic Agonist Functional Assays

Subtype selectivity of *trans*‐PAI was studied by comparing the amplitude of fluorescence calcium imaging responses of cells expressing M2‐GqTOP and M1 mAChRs. Human M2‐GqTOP mAChR was transfected as described in Riefolo et al. 2019.^[^ ^37^ ^]^ Human M1 mAChR (Addgene) was transfected with X‐tremeGENE 9 DNA Transfection Reagent (Roche Applied Science) following the manufacturer's instructions. The day after transfection, cells were harvested with accutase (Sigma‐Aldrich) and seeded onto 16‐mm glass coverslips (Fisher Scientific) pretreated with collagen (Sigma‐Aldrich) to allow cell adhesion. Preconfluent cultures were used for experiments between 48 and 72 h after transfection. In vitro single‐cell calcium imaging was performed as described in Riefolo et al. 2019,^[^ ^37^ ^]^ pre‐incubating cells with OGB‐1AM (10 × 10^–6^ m for 30 min) as calcium indicator. The subtype selectivity of 3 and 10 × 10^–12^ m *trans*‐PAI emerged by comparing the amplitude of fluorescence calcium imaging responses of cells expressing M1 (3 × 10^–12^ m, *n =* 232 cells; 10 × 10^–12^ m, *n =* 232 cells; IPX, *n =* 258) or M2‐GqTOP (3 × 10^–12^ m, *n =* 70 cells; 10 × 10^–12^ m, *n =* 200 cells; IPX, *n =* 300) mAChRs. Cells transfected with M2 mAChR gave significantly higher responses than M1 mAChR expressing cells (33% for M2 compared to 8% for M1). Data were normalized over the maximum response obtained with the nonselective agonist Iperoxo (IPX) at saturating concentration of 30 × 10^–12^ m (*t*‐test of two samples assuming equal variances. *p* = 1.58 × 10^−3^).

A preliminary comparison between mAChR subtypes was obtained using an in vitro muscarinic agonist functional assay (Eurofins GTPgammaS assay) which allows us to estimate possible activation of M1 and M2 receptors (as expressed in the Eurofins specifications). In particular, different concentrations of the active isomer *trans*‐PAI were tested on human recombinant M1 and M2 receptors expressed in CHO‐K1 (Chinese hamster ovary) cells. Compound agonism is defined by quantitation measured of bound [^35^S]GTP*γ*S. PAI was preincubated with 0.045 mg ml^−1^ membranes and 3 × 10^–6^ m GDP for 30 min at 30 °C in modified HEPES buffer pH 7.4 with SPA beads are added for another 60 min at 30 °C. The reaction is then initiated by the addition of 0.3  × 10^–9^ m [^35^S]GTP*γ*S for 30 min. *Trans*‐PAI‐induced increase of [^35^S]GTP*γ*S binding by 50% or more (≥50%) relative to 1  × 10^–9^ m (M1) or 1 × 10^–6^ m (M2) oxotremorine M responses indicates possible M1 or M2 agonist activity, as indicated in the Eurofins assay specifications. Experiments were performed in duplicates (*n* = 2) and were accepted in accordance with Eurofins Quality Control Unit's validation standard operating procedure.

### Statistical Analysis

In vitro and in vivo oscillatory frequency, firing rate (FR) during the Up‐state, PSD of oscillatory activity values are reported as mean ± SEM. All the oscillatory frequency and FR during the Up‐state analyses in brain slices of Iperoxo (IPX), PAI dose‐response curves, and 200  × 10^–9^ m PAI photoconversion experiments were performed with one‐way ANOVA test (Brown‐Forsythe and Welch test, unpaired *t* with Welch's correction) of GraphPad Prism 9. The analyses in brain slices between *trans‐* and *cis‐*PAI oscillatory frequency and FR during the Up‐state values were performed with multiple *t*‐test (Mann‐Whitney) of GraphPad Prism 9. All the in vivo statistical analyses of oscillatory frequency and firing rate (FR) during the Up‐state were performed with one‐way ANOVA test (repeated measures [RM], Geisser–Greenhouse correction—no sphericity—and uncorrected Fisher's LSD) of GraphPad Prism 9. PSD statistical analysis in vivo was performed with Friedman test and the Wilcoxon post‐hoc tests corrected for multiple comparisons of MATLAB R2020b. Calcium imaging analyses of 3 and 5 independent cell preparations expressing M1 and M2‐GqTOP mAChRs, respectively, were first normalized over the basal fluorescence of the cells. The fluorescence amplitudes of cellular PAI‐induced calcium oscillations were normalized over IPX responses at the saturating concentration of 30 × 10^–12^ m, reported as mean ± SEM, and compared using a *t*‐test of two samples (assuming equal variances) using Origin Pro 8.5.

## Conflict of Interest

The authors declare no conflict of interest.

## Supporting information

Supporting Information

## Data Availability

The data that support the findings of this study are available from the corresponding author upon reasonable request.
