# Supplementary material for: Control of Brain State Transitions with a Photoswitchable Muscarinic Agonist
Source: Adv Sci (Weinh). 2021 May 21;8(14):2005027. doi: 10.1002/advs.202005027 (PMC8292914; doi:10.1002/advs.202005027)
Supplement: Supplementary file 1 — Supporting Information [file ADVS-8-2005027-s001.pdf]

WILEY-VCH

© 2021 Wiley-VCH GmbH

ADVANCED SCIENCE
Open Access

Supporting Information

for *Adv. Sci.*, DOI: 10.1002/advs.202005027

Control of brain state transitions with a photoswitchable muscarinic agonist

Almudena Barbero-Castillo, Fabio Riefolo, Carlo Matera, Sara Caldas-Martínez, Pedro Mateos-Aparicio, Julia F. Weinert, Aida Garrido-Charles, Enrique Claro, Maria V. Sanchez-Vives, and Pau Gorostiza**

Supporting Information

Control of brain state transitions with a photoswitchable muscarinic agonist

Almudena Barbero-Castillo[†]*, Fabio Riefolo*[†]*, Carlo Matera, Sara Caldas-Martínez, Pedro Mateos-Aparicio, Julia F. Weinert, Aida Garrido-Charles, Enrique Claro, Maria V. Sanchez-Vives*[5,*]*, and Pau Gorostiza*[5,*]

1. **Competition Binding and *in vitro* GTPgammaS assays**

2. Dose-Response curves of *trans*- and *cis*-PAI in ferret brain slices

3. Autocorrelograms

4. *Trans*-to-*cis* photoconversion and pharmacological competition of PAI effects *in vivo*

1. Competition binding and *in vitro* GTPgammaS assays

1.1 Competition binding experiments

1.1.1 Materials and Methods

Iperoxo (IPX) and Phthalimide-Azo-Iperoxo (PAI) were preliminarily assayed for their affinity to muscarinic receptors (mAChRs) by competition binding experiments in whole cortex of 3–4-month-old female Wistar rat brain membrane, which contained a high density of all the subtypes of mAChRs.[1] [^{3}H]Quinuclidinyl benzilate ([^{3}H]QNB) is a muscarinic antagonist without subtype selectivity, which binds muscarinic receptors with high selectivity. [^{3}H]QNB is recognized to be excellent for such binding experiments,[2,3] and we performed them in order to test if IPX and PAI have the potential to modulate cortical brain states. Specific binding of IPX and PAI was defined by testing concentrations ranging from 10^{-9} to 10^{-4} M, and derivatizing the raw disintegrations per minute (dpm) data from the scintillation counter to obtain the total radioactivity values.[1]

1.1.2 Results

Competition binding experiments can show that both IPX and PAI have an interesting high binding affinity for mAChRs orthosteric site. IPX was found to have an IC_{50} of 1.5 µM, *trans*-PAI of 47 nM and *cis*-PAI of 118 nM (**Fig. S1.1**). Such preliminary results encouraged us to investigate further our muscarinic compounds activity on the dynamics of the isolated V1 cortical slices.

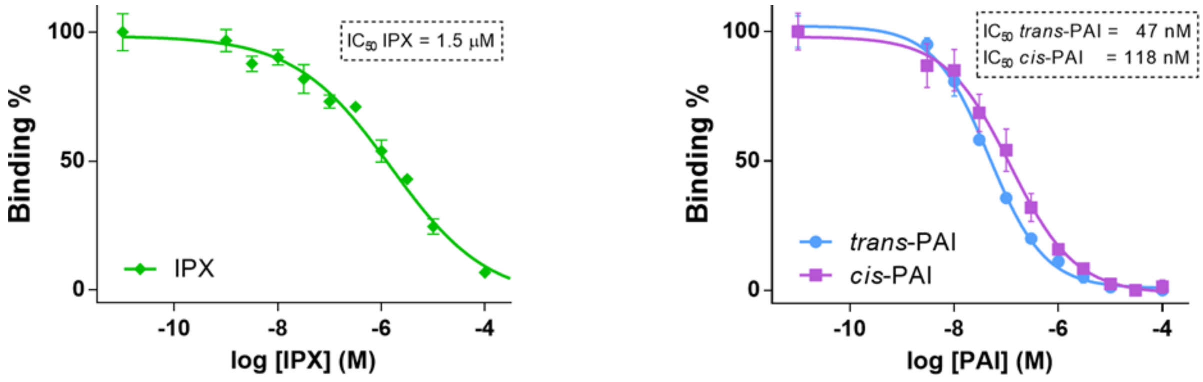

Figure S1.1. Competitive binding experiments of IPX and PAI to Wistar rats' whole cortex containing all the mAChRs. Competitive binding experiments of IPX and PAI to Wistar rats' whole cortex containing all the mAChRs. Competition for specific binding of 200 pM [^{3}H]QNB to 3-4 months-old female Wistar rats brain membranes (whole cortex) containing high density of all the five mAChRs by IPX and PAI (n = 4 for each isomer). Data points are reported as mean ± SEM and were fitted using the "log(inhibitor) vs. normalized response - Variable slope (four parameters" function in GraphPad Prism 6).

1.2 *in vitro* GTPgammaS assay

1.2.1 Materials and Methods

The detailed description of GTPgammaS assay's materials and methods are reported in the main text (Experimental Section).

1.2.2 Results

An *in vitro* muscarinic agonist functional assay (Eurofins GTPgammaS assay) was used to preliminarily estimate possible activation of M1 and M2 receptors (as expressed in the Eurofins specifications). The active *trans*-PAI isomer showed M2 preference in a wide range of concentrations (1 nM to 10 µM). M2 selectivity was demonstrated using a functional assay (calcium imaging) in cells overexpressing M1 and M.2 receptors (**Fig. S1.2**).

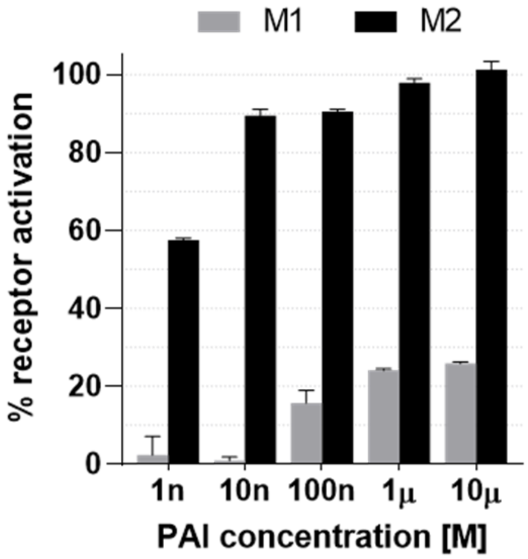

Figure S1.2. ***In vitro* GTPgammaS functional assay for screening the *trans*-PAI-induced M1 and M2 mAChRs activation.** Different concentrations (1, 10, 100 nM, and 1, 10 µM) of the active *trans*-PAI isomer_were tested on human recombinant M1 and M2 receptors expressed in CHO-K1 (Chinese hamster ovary) cells. *Trans*-PAI agonism is identified by quantitation measured of bound [^{35}S]GTPγS. The increase of [^{35}S]GTPγS binding by 50 percent or more (≥50%) relative to 1 mM for M1 or 1 µM for M2 oxotremorine M responses indicates possible agonist activity of t*rans*-PAI. Experiments were performed in duplicates (n=2) and were accepted in accordance with Eurofins Quality Control Unit's validation standard operating procedure.

2. Dose-Response curves of *trans*- and *cis*-PAI in ferret brain slices

In a total of n=12 ferret brain slices, the baseline activity (characterized by SO) was recorded as a control, prior to bath-application of increasing PAI concentrations (10 nM, 100 nM, 300 nM, and 1 µM, n=6 ferret brain slices for each PAI form, *trans* and *cis*).

100, 300 nM, and 1 µM *trans*-PAI significantly increased the oscillatory frequency from control condition (0.58 ± 0.06 Hz) (**Fig. 2** and **Fig. S2**). In particular, 100 nM to 0.87 ± 0.11 Hz, 300 nM to 1.66 ± 0.10 Hz, and 1 µM to 1.87 ± 0.13 Hz.

300 nM and 1 µM *trans*-PAI also significantly decreased the FR during the Up-states from control condition (0.98 ± 0.11 a.u.) to 0.51 ± 0.08 a.u. and 0.37 ± 0.03 a.u. respectively (**Fig. 2** and **Fig. S2**).

Differently, *cis*-PAI at 100 nM and 300 nM did not significantly alter the spontaneous oscillatory frequency observed in control experiments (0.48 ± 0.037 Hz), in contrast to the obvious modulation in oscillatory activity obtained with 100 nM and 300 nM *trans*-PAI (**Fig. 2** and **Fig. S2**). Only at concentrations as high as 1 µM did *cis*-PAI significantly alter the Up- and Down-state sequence in comparison to the control, increasing the oscillatory frequency to 1.32 ± 0.27 Hz (**Fig. 2S**).

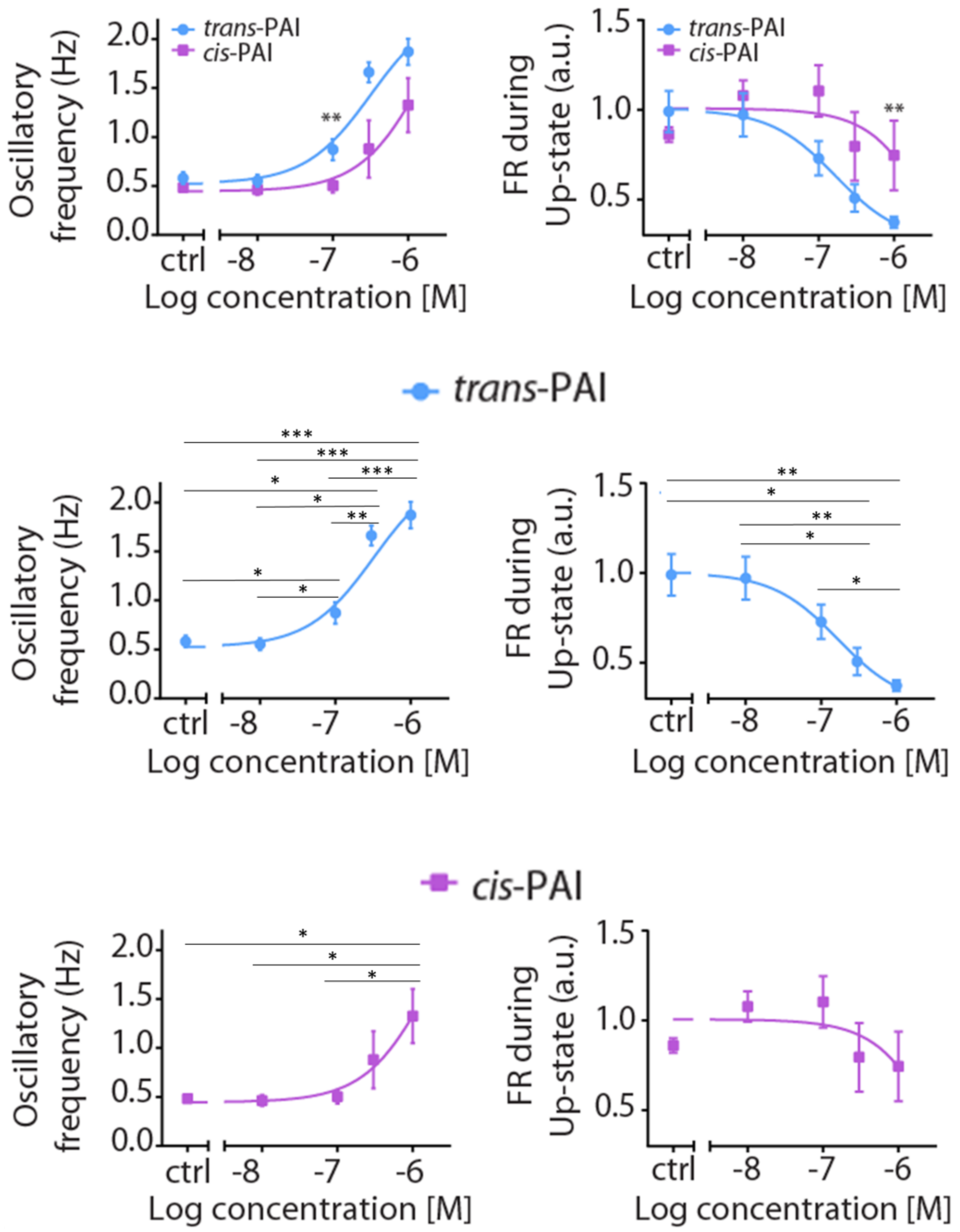

Figure S2. Effect on SO of mAChRs activation by trans-PAI (dark-relaxed) and cis-PAI (pre-illuminated with UV) in ferret brain slices. Oscillatory frequency (Hz) and FR during the Up-states (a.u.) of the PAI isomers, *trans*- (blue, n = 6 ferret brain slices) and *cis*-PAI (pink, n = 6 ferret brain slices) at different concentrations. Data are reported as mean ± SEM. Above, the panel **E** of **Fig.2** is reported again to facilitate the discussion. Significant differences between *cis*- and *trans*-PAI (**p-value < 10^{-2}) are observed in the oscillatory frequency at 100 nM and in the FR at 1 µM. Analyses were performed with with multiple t-test (Mann-Whitney). In the middle and below, the same *trans*-PAI (blue) and *cis*-PAI (pink) values of the oscillatory frequency (Hz) and FR during the Up-states (a.u.) are separately reported in order to show the significant differences at different concentrations. Analyses were performed with one-way ANOVA test (Brown-Forsythe and Welch test, unpaired t with Welch's correction). p-values: * < $5 \cdot 10^{-2}$; ** < 10^{-2}; *** < 10^{-3}. These experiments are aimed at estimating the concentration of effective photoswitching of cortical oscillations, which was set at 200 nM in subsequent experiments (**Figs. 3** and **4**).

3. Autocorrelograms

3.1 Autocorrelograms at 200 nM of PAI (switching of PAI activity)

PAI (200 nM) was first applied in its less active isomer (*cis*-PAI, pre-irradiation with 365 nm light). The activity of *cis*-PAI at 200 nM (pink line) in cortical slices evokes very slight changes in terms of oscillatory activity with respect to the basal control situation (black line; before PAI application, as is shown in the autocorrelograms (**Fig. S3.1**). After PAI application and white light (WL) illumination, PAI switches to its more active *trans* form (blue line), inducing obvious changes of the oscillatory activity (**Fig. S3.1**). The autocorrelograms were obtained by analyzing LFP from one channel.

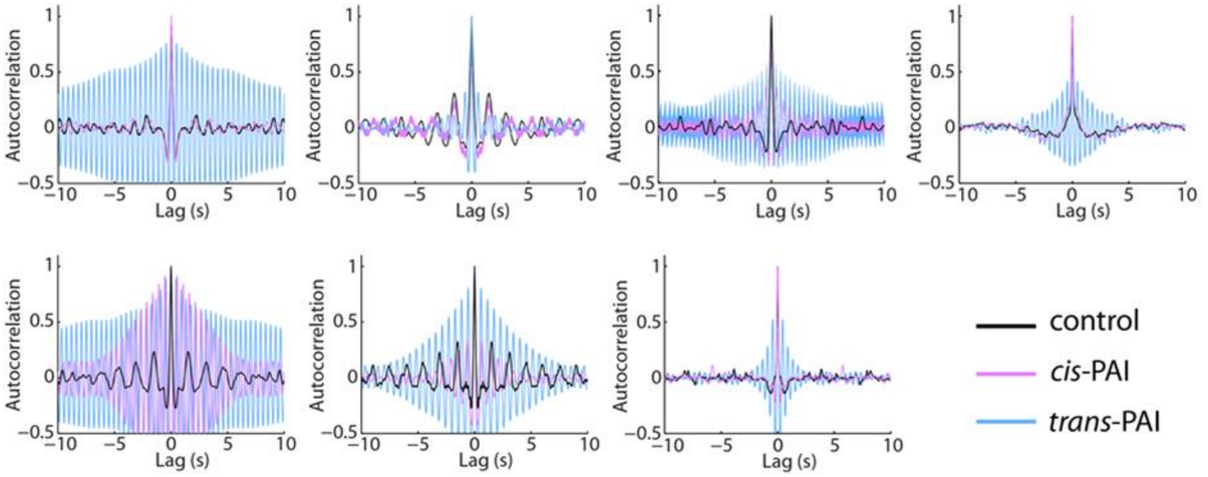

Figure S3.1 PAI can light-modulate the neuronal oscillatory activity in cortical ferret slices. Autocorrelograms of the rhythmicity of the neuronal oscillatory activity in basal condition (without PAI application, black line), under 200 nM *cis*-PAI application (pink line) and during white light irradiation (*trans*-PAI, blue line).

3.2 Autocorrelograms at 100 nM and 1 μM of PAI

The activity of PAI at 100 nM and 1 μM in cortical slices did not strongly differ between trans (**Fig. S3.2**) and *cis* (**Fig. S3.3**) in terms of oscillatory activity. 100 nM applications of *trans* and *cis* did not induce significant changes in neuronal firing in comparison to the basal control situation (black line), as is shown in the autocorrelograms (**Fig. S3.2** and **S3.3**). At 1 μM, both PAI isomers produce changes in the oscillatory activity (**Fig. S3.2** and **S3.3**). The autocorrelograms graphs are obtained by analyzing LFP from one channel.

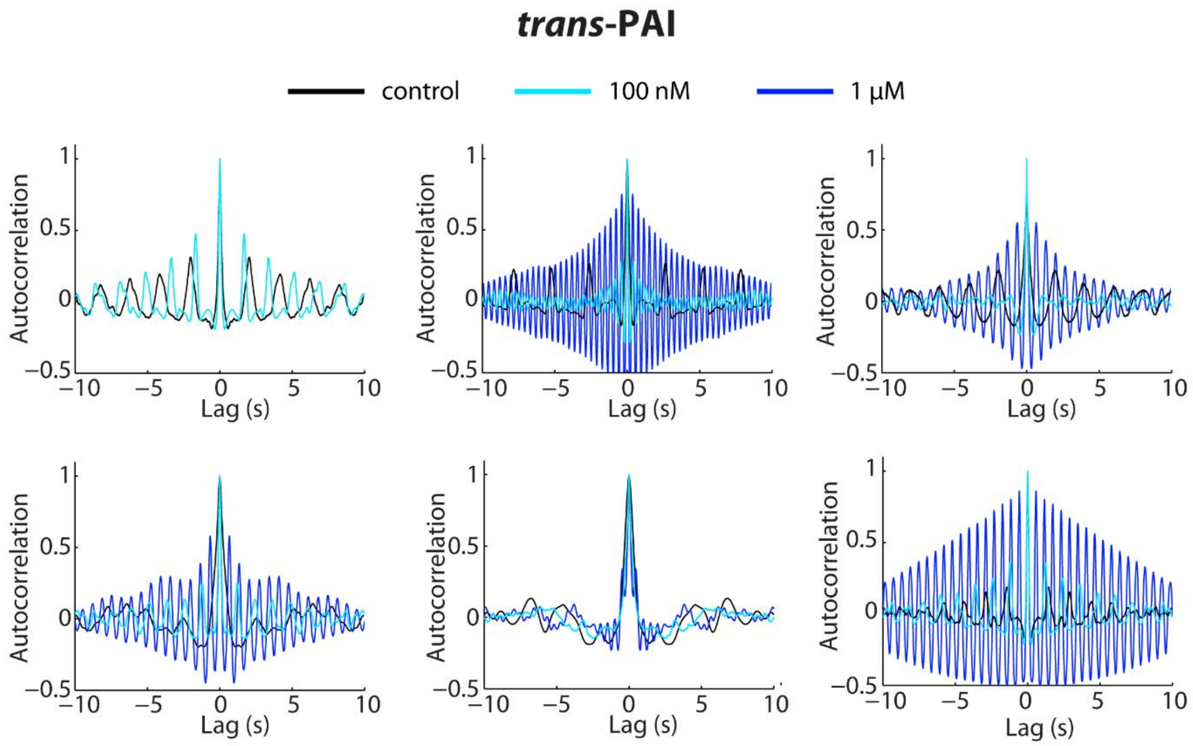

Figure S3.2 Autocorrelograms of the LFP illustrating the neuronal oscillatory activity in basal condition (without PAI application, black line), at 100 nM and 1 μM of *trans*-PAI (light and dark blue lines) applications in six cortical slices.

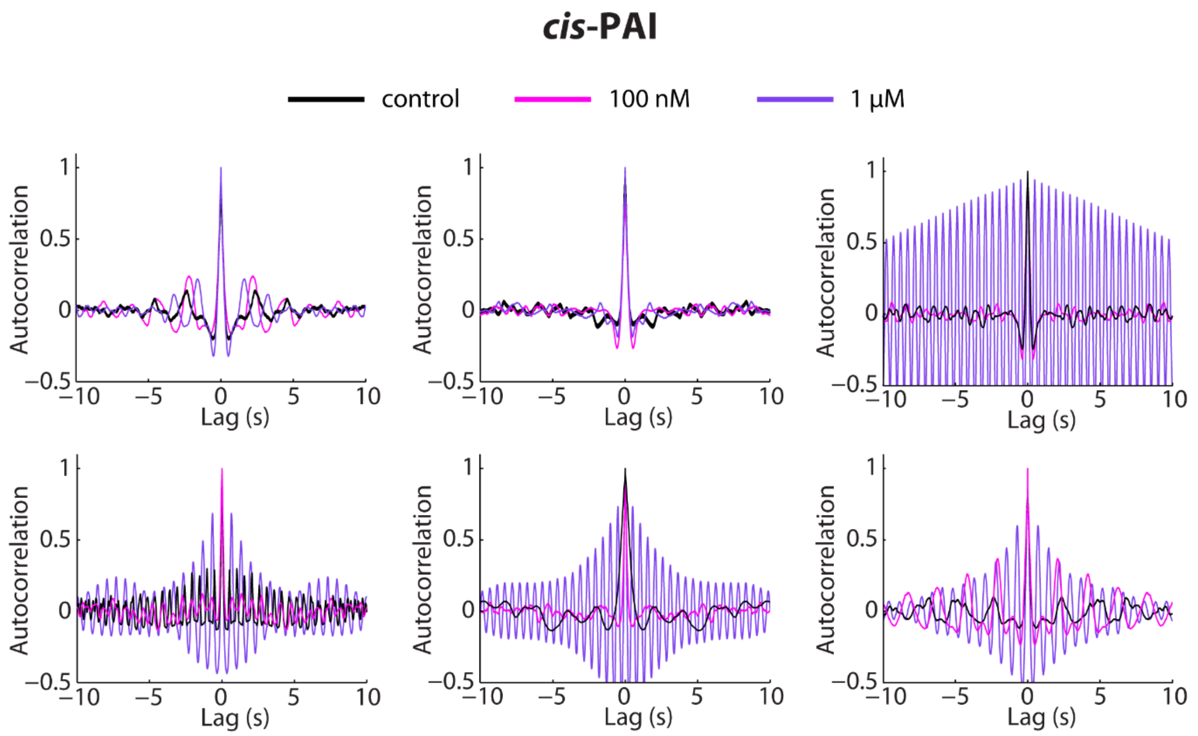

Figure S3.3. Autocorrelograms of the rhythmicity of the neuronal oscillatory activity in basal condition (without PAI application, black line), at 100 nM and 1 μM of *cis*-PAI (pink and purple lines) applications in six cortical slices.

4. *Trans*-to-*cis* photoconversion and pharmacological competition of PAI effects *in vivo*

Two separated different approaches were used to try to revert the excited oscillatory frequency induced by *trans*-PAI *in vivo:* (a) the photoconversion (*n*=4) *in situ* with a powerful 365 nm light source, and (b) the pharmaco-competition by applying its inactive *cis* isomer (*n*=4). According to our description in **Fig. 4**, application of 1 µM *cis*-PAI did not alter the oscillatory frequency (from 0.55 ± 0.07 Hz under control and 0.55 ± 0.09 Hz under *cis*-PAI) (**Fig. S4A**), while illumination with WL increased the oscillatory frequency (from 0.55 ± 0.09 Hz under *cis*-PAI to 0.64 ± 0.08 Hz upon WL illumination) (**Fig. S4A**). *Trans*- to *cis*-PAI photoconversion with a very powerful 365 nm lamp (Spectroline, Spectronics FC-100-F, 230V, 365nm 100 W Spot Bulb) did not produce a significant decrease in the oscillatory frequency *in vivo* (from 0.64 ± 0.08 Hz to 0.59 ± 0.05) (**Fig. S4A**, indicated with a violet flashlight). We reasoned that this could be due to (1) limited photoisomerization of PAI caused by poor tissue penetration of UV light, and (2) intrinsic pharmacological irreversibility of PAI effects caused by e.g. the intracellular signaling pathway of M2 mAChRs, by the neural circuits involved in cortical oscillations, or other reasons. We ruled out the second possibility with the following experiment i*n vivo*. We applied 200 nM *cis*-PAI and observed again a significant increase of the OF upon illumination with white light (indicated with the first yellow flashlight in **Fig. S4B** and labeled $2 \cdot 10^{-7}$ M). We subsequently applied 1 µM *cis*-PAI and observed a significant decrease in the OF back to the control level (indicated with the second violet bar in **Fig. S4B** and labelled 10^{-6} M). This result demonstrates that outcompeting *trans*-PAI (active isomer) with a 5-fold higher concentration of *cis*-PAI (less active isomer) fully reverts the effect of *trans*-PAI in cortical oscillations *in vivo*. The action of PAI is thus reversible pharmacologically and the fact that UV light cannot restore the OF to control levels (**Fig. S4A**) must be attributed to the limited penetration of UV light.

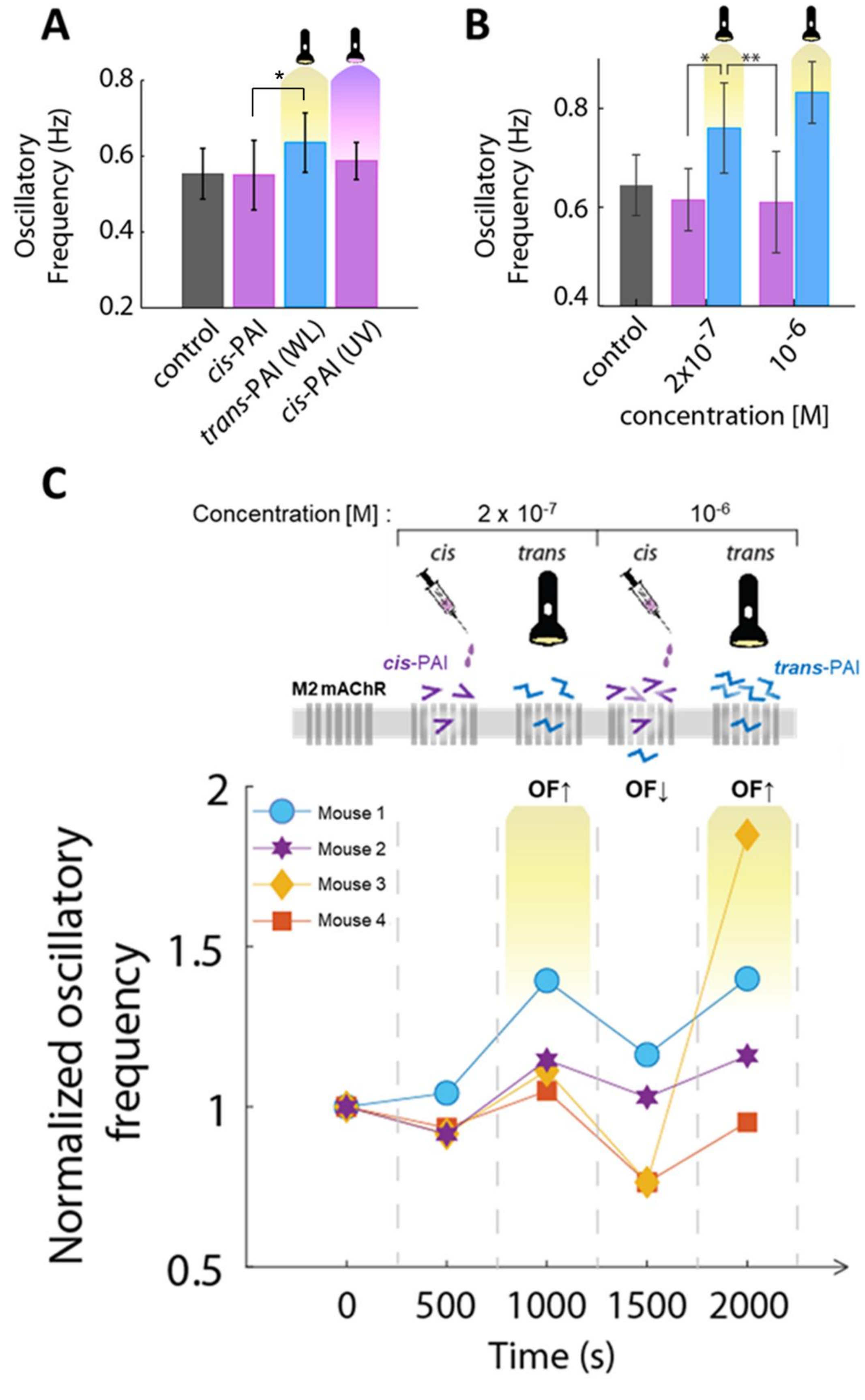
A
Oscillatory Frequency (Hz)
0.8
0.6
0.4
0.2
*
control
cis-PAI
trans-PAI (WL)
cis-PAI (UV)
B
Oscillatory Frequency (Hz)
0.8
0.6
0.4
*
**
control
2x10-7
10-6
concentration [M]
C
Concentration [M] :
2 x 10-7
10-6
cis
trans
cis
trans
cis-PAI
trans-PAI
M2 mAChR
OF↑
OF↓
OF↑
Mouse 1
Mouse 2
Mouse 3
Mouse 4
Normalized oscillatory frequency
2
1.5
1
0.5
0
500
1000
1500
2000
Time (s)

Figure S4. Reversibility of the photocontrol of cortical oscillations with PAI *in vivo* and cause of its limitations. (**A**) The increase in oscillatory frequency elicited by photoconverting *cis*-PAI to *trans*-PAI cannot be reverted with UV light. We applied 1 µM *cis*-PAI, photoconverted it to *trans*-PAI with White Light (WL, yellow flashlight) and observed a significant increase in oscillatory frequency. This effect was not reversible upon illumination with a UV lamp (100 W, indicated by violet flashlight) (n = 4 mice). **p-value* $<5\cdot10^{-2}$. (**B**) The excited state evoked by photoisomerizing *cis*-PAI to *trans*-PAI is fully reversible by subsequent administration of *cis*-PAI. Pharmacological competition between 200 nM *trans*-PAI, after photoconversion with WL (indicated by first yellow flashlight), and 1 µM *cis*-PAI (n=4 mice different from **A**). The application of 1 µM *cis*-PAI significantly decreased the oscillatory frequency to that of the control condition. The lack of photo-reversibility in (**A**) is thus due to the poor penetration of UV light in brain tissue. **p-value* $<5\cdot10^{-2}$ and ***p-value* $< 10^{-2}$. Color code: *cis*-PAI = violet and *trans*-PAI WL = blue. Data of panel A and B are reported as mean ± SEM. Analyses were performed with one-way ANOVA test (repeated measures [RM], Geisser-Greenhouse correction –no sphericity- and uncorrected Fisher's LSD). (**C**) Time-course of normalized oscillatory frequency of the 4 individual mice during the *trans*/*cis*-PAI pharmacological competition experiments. Diagrams indicate an inactive muscarinic receptor in the presence of 10^{-7} M *cis*-PAI (depicted as a violet V-shape, added with a syringe), an activated receptor in the presence of 10^{-7} M *trans*-PAI (blue Z-shape, photoisomerized with a white flashlight), and again an inactive receptor in which the addition of 10^{-6} M *cis*-PAI (violet V-shape, indicated with a syringe) has outcompeted *trans*-PAI from the previous step.

Supporting Information References

[1] E. Claro, Biochem. Mol. Biol. Educ. 2006, 34, 428–431.

[2] H. I. Yamamura, S. H. Snyder, Proc. Natl. Acad. Sci. U. S. A. 1974, 71, 1725–9.

[3] J. Sallés, M. A. Wallace, J. N. Fain, J. Pharmacol. Exp. Ther. 1993, **264.**
